# Chronic Atrazine Exposure Beginning Prenatally Impacts Liver Function and Sperm Concentration With Multi-Generational Consequences in Mice

**DOI:** 10.3389/fendo.2020.580124

**Published:** 2020-11-26

**Authors:** Alesia P. Harper, Bethany J. Finger, Mark P. Green

**Affiliations:** School of BioSciences, University of Melbourne, Parkville, Melbourne, VIC, Australia

**Keywords:** atrazine, endocrine disrupting chemical, reproduction, testis, embryo, liver, fatty acid

## Abstract

Atrazine is a commonly used herbicide frequently detected in waterways and drinking water around the world. Worryingly, atrazine is an endocrine and metabolic disruptor but there is a lack of research regarding the effects of long-term exposure beginning *in utero*. In this study we investigated how chronic exposure to atrazine (5 mg/kg bw/day) in drinking water from E9.5 until 12 or 26 weeks of age affected metabolic and reproductive characteristics in male mice. We then examined whether mating these males to unexposed females altered *in vitro* embryo characteristics. Atrazine exposure caused a decrease in liver weight and changes in both liver and testis gene expression, specifically in genes involved in lipid uptake and fatty acid metabolism in the liver, as well as androgen conversion in the testis. Notably, atrazine exposure decreased epididymal sperm concentration and subsequent embryo cell numbers generated from the 12-week cohort males. Collectively, these data suggest that atrazine exposure, beginning prenatally, affects both metabolic and reproductive characteristics, and highlights the importance of assessing atrazine effects at different life stages and over multiple generations. The continued widespread use of atrazine warrants further studies, as it is essential to understand the health risks for all species, including humans.

## Introduction

Pesticides play an important role in improving agricultural production. There are however concerns that these chemicals may have adverse effects on human health and on the environment (1–3), as many pesticides are known to be endocrine disrupting chemicals (EDCs) (2, 3), yet, the safety of these chemicals is not well established, despite their increasing use (1, 4). One pesticide of concern is atrazine, due to its widespread use, slow biodegradation, and endocrine disrupting abilities (1).

Atrazine (2-chloro-4-ethylamino-6-isopropylamino-1,3,5-triazine, ATZ) is one of the most commonly used herbicides in agriculture, with more than 3,000 and 32,500 tonnes administered per year in Australia (5) and the United States respectively (6). Atrazine is used to control the growth of broadleaf weeds and annual grasses and is frequently used on crops such as corn, sugarcane and sorghum, as well as on urban golf courses (5). Atrazine is highly persistent in the environment, being detected up to 40 μg/L in rainwater (7), and with a half-life in surface water of over 100 days (8), making it one of the most commonly detected contaminants in waterways (5, 9). Based on consistent groundwater contamination and its potential risks to human health atrazine was banned in 2003 in the European Union (EU) (10), although atrazine is still very heavily used in many countries (4, 7).

Human exposure to atrazine is most likely to occur through drinking water, with drinking concentrations between 0.01 and 5 μg/L (11), irrespective of whether they live in an urban or rural location. The detection of atrazine in human bodily fluids, including follicular fluid, sperm fluid and cervical mucus (12), as well as studies showing an association with the birth of preterm and small-for-gestational-age babies (13, 14), supports its potential to negatively affect human reproductive health, although other studies report inconsistent findings (15). Effects on the reproductive system are corroborated by evidence from animal studies that shows atrazine has endocrine disrupting effects across multiple animal classes, including amphibians (16, 17), fish (17, 18) reptiles (17) and rodents (19, 20).

The mechanism by which atrazine exerts its effects within the body is not entirely understood. Initially atrazine was determined to be anti-androgenic, to decrease levels of both testosterone and the more potent androgen dihydrotestosterone (DHT) (21). Atrazine acts by decreasing the expression of 5α-reductase, which is the enzyme responsible for the conversion of testosterone to DHT (22). Atrazine is also known to have oestrogenic effects by increasing *Cyp19a1* expression that encodes the enzyme aromatase, which converts testosterone to oestradiol (21). These effects are evident *in vivo* in amphibians (16) and rats (20), as well as *in vitro* in human oestrogen-sensitive tissues (23). Imbalances in these hormones can have a direct effect on the physiology of reproductive organs (24), and exposure to atrazine has caused aggregation and increases in foetal Leydig cell number leading to reduced serum testosterone in post-pubertal males (25), delayed pubertal onset (19), alterations to hormone levels (24, 26), and reduced sperm viability in male rodents (19, 24, 25, 27).

More recently atrazine was identified to have obesogenic effects in rodents; i.e., it can perturb metabolic activity by interfering with mitochondrial function (28). Linked to this, early life exposure to EDCs, including atrazine, is believed to be a causal factor that may explain the increasing incidence of hepatic steatosis (fatty liver disease) in humans (29). Few studies in mammals have however investigated the effect of atrazine on metabolism and liver function (26, 30–33), especially with respect to the effects of chronic prolonged exposure (longer than 3 months) (28), and no studies have examined effects of exposure prior to birth on offspring metabolism. This is surprising, given the main use of atrazine as a herbicide in agriculture is to disrupt the photosynthetic metabolism of plants (34). Interestingly, in the USA there is a considerable amount of overlap in areas of high atrazine use and obesity prevalence in humans (28, 35). This association was confirmed in rodent models, in which chronic exposure to environmentally relevant atrazine doses (30 µg/kg bw/day) induced body weight gain, insulin resistance and hepatic steatosis with a high fat diet (28). Notably, these effects on metabolism are likely to impact upon reproductive function, as these regulatory pathways are often overlapping (36).

The current acceptable daily intake (ADI) for atrazine of 0.005 mg/kg bw in Australian drinking water is based on a no-observed-effect-level (NOEL) of 0.5 mg/kg bw/day from a rodent study incorporating an added safety factor of 100 (37). Research in our lab has determined that exposure from weaning at 4 weeks of age to 12 weeks of age to atrazine in drinking water, using a dose 10-fold higher (5 mg/kg bw/day) than the NOEL, resulted in reproductive and metabolic phenotypic changes in male mice (33). Studies using similar atrazine dosages (1 to 125 mg/kg bw/day) administered in food have identified, at the higher concentrations, reduced body and testis weight, increased percentage of abnormal sperm in male rats (24). In light of the Developmental Origins of Health and Disease (DOHaD) hypothesis (38), which determined that environmental stressors occurring in the peri-conceptional period or gestation can alter the development and function of organs, and pre-dispose individuals to diseases or health implications later in life (38), we decided, as a proof of concept, to utilise a similar experimental design to our previous study but starting atrazine exposure *in utero*, just prior to organ formation, at gestation day 9.5, and extending the exposure to be chronic; early maturity and breeding age (12 weeks of age) or well in to maturity (6 months of age). Thus, we aimed to investigate if prolonged exposure to an atrazine dose, starting *in utero* through to adult life, would result in similar or more pronounced phenotypic changes, both in terms of metabolic (obesity, liver characteristics and disease - steatosis) and reproductive changes (testis function and morphology, as well as sperm characteristics). Ultimately, we hypothesised that atrazine, at the current dosage, would negatively affect organ development and gene expression of the liver and testis, and this would result in perturbations in metabolic homeostasis, as well as sperm parameters and subsequent embryo characteristics, instigating multi-generational effects.

## Materials and Methods

### Laboratory Chemicals and Consumables

All chemicals used were purchased from Sigma-Aldrich (Castle Hill, NSW, Australia), unless otherwise specified. Culture media were purchased from Vitrolife (Göteborg, Sweden).

### Animals and Experimental Design

All mice were kept in the School of BioSciences animal housing facility at the University of Melbourne, maintained under a 12 h light: 12 h dark lighting regimen and fed *ad libitum* a soy-free diet (Specialty Feeds, Perth, WA, Australia) to avoid the effects of phytoestrogens. Six-week old female C57BL/6J (Melbourne Bioresources Platform) mice were mated overnight with 12-week-old C57BL/6J males. Mating was determined by presence of a copulatory plug at day 0.5 of pregnancy. Pregnant females were then individually housed and randomly allocated to one of two treatment groups: Control (CON, n = 5) and Atrazine (ATZ, n = 5). Atrazine was initially dissolved in DMSO, prior to being added in distilled drinking water (< 0.5% DMSO v/v). Pregnant females (n = 5 per group) were administered from day 9.5 of gestation either a vehicle treatment (< 0.5% DMSO, CON) or atrazine treatment (5 mg/kg bw/day, atrazine), based on our previous studies (33) and standard water consumption rates. The selected dose is 10-fold higher than the NOEL used to calculate the acceptable daily intake (ADI) for drinking water in Australia (37). Oral administration of this dose is known to expose mouse foetuses to a similar *in utero* concentration to that measured in maternal plasma (39). Pups were weaned at four weeks of age, as per standard animal husbandry practices, with only male pups utilised for this study. These males were housed individually with continued exposure to the treatment water (CON or ATZ). Within each treatment littermates were split up and maintained until either i) 12 weeks of age (n = 15) or ii) 26 weeks of age (n = 20). A soy-free diet and water were provided *ad libitum* and their consumption was monitored for six males from each treatment group for five weeks. Body weights were recorded weekly. All experiments were approved by the University of Melbourne Animal and Ethics committee (AEC 1513481.5) in accordance with the Australian National Health and Medical Research Council guidelines (40).

### Embryo Culture Studies

At 11 and 25 weeks of age males (n > 5, at least one from each litter) from each treatment group, and age cohort, were randomly selected for mating. Three-week old C57BL/6J female mice (n > 20 per treatment per age cohort) were superovulated using a standard protocol (41), with an intraperitoneal injection of 0.25 IU/g pregnant mare serum gonadotrophin (PMSG; Folligon, Intervet, Bendigo, Australia) followed 48 h later by 0.25 IU/g human chorionic gonadotrophin (hCG; Chorulon, Intervet). Individual males and females were housed overnight for mating and 2 h post-hCG injection, pronucleate oocytes (2PN) were recovered from female tracts and placed in G-MOPS handling medium supplemented with 5 mg/ml human serum albumin (GMOPS+; Vitrolife, Göteborg, Sweden). Pronucleate oocytes were denuded of cumulus cells *via* incubation in GMOPS containing 300 IU/ml hyaluronidase for 20 s (bovine testes, type IV; Sigma-Aldrich) followed by washing in GMOPS+. Denuded pronucleate oocytes were immediately washed in GMOPS+ and transferred in groups of 10 to 20 µl drops of G1 media (Vitrolife) under 3.5 ml paraffin oil (Ovoil) for 72 h at 6% CO_2_, 5% O_2_ and 89% N_2_ at 37°C as previously detailed (42). Embryos were then assessed for development and transferred to pre-equilibrated G2 media (Vitrolife) for a further 48 h. After a total 96 h culture, pre-implantation embryos were assessed for development stage and subjected to differential staining to identify the allocation of cells to the inner cell mass (ICM) and the trophectoderm (TE) of blastocysts, as described previously (41). Briefly, blastocysts were placed in 0.5% v/v pronase (Sigma-Aldrich) until the zona pellucida disbanded, followed by washing in GMOPS+ for 5 min. Embryos were then incubated in 10 mM 2,4,6-trinitrobenzene sulfonic acid (Sigma-Aldrich) for 10 min then washed in GMOPS+ for 5 min, before a 10 min incubation in 0.1 mg/ml anti-dinitrophenol (Sigma-Aldrich). Blastocysts were subsequently washed for 5 min in GMOPS+, then incubated in 10% v/v guinea pig serum with 25 mg/ml propidium iodide (IMVS, Adelaide, Australia) for 5 min. Blastocysts were transferred to 0.1 mg/ml bisbenzimide (Hoechst, 33342) in 10% v/v ethanol for 15 min, washed in GMOPS+ and finally mounted in glycerol on glass slides under coverslips (Thermo Fisher, Scoresby, VIC, Australia). Cells were visualised and photographed using a fluorescent microscope (Nikon Eclipse TS100) equipped with a Nikon Digital Sight DS-L2 camera (Nikon, Tokyo, Japan). Cell numbers were counted using ImageJ Version 1.47 (43), by a technician blinded to treatment groups. Five biological embryo culture replicates per age cohort were undertaken.

### Post-Mortem Tissue Collection

Mice were killed *via* cervical dislocation at i) 12 weeks of age (n = 15 males from five litters) or ii) 26 weeks of age (n = 20 males from five litters). At post-mortem body weight was recorded, the epididymides were immediately dissected out and transferred to a tissue culture dish (Thermo Fisher) containing G-MOPS+ at 37°C. An epidydimal sperm count and live:dead sperm stain was performed, as described below. The liver, testes, seminal vesicles, perigonadal fat, and retroperitoneal fat were removed and weighed. Samples of liver and testis were snap frozen in liquid nitrogen and stored at -80°C for gene expression analysis. Additional samples of liver and testis were fixed in 4% (v/v in 0.9% saline) paraformaldehyde (PFA) for histological analysis.

### Histology of Liver and Testis Sections

Sections of liver and testis fixed in 4% paraformaldehyde (v/v) were washed in 0.9% saline and stored in 70% ethanol at 4°C (44). These tissues were then embedded in paraffin wax, sectioned at 7 µm thickness and stained with Harris Haemotoxylin & Eosin Y (H & E) following standard protocols and as previously described (45). Sections were visualised using an Olympus BX51 Microscope (Olympus, Tokyo, Japan) and images were captured using an Olympus DP70 Camera (Olympus). Liver and testis sections were assessed at x 20 magnification for the presence of gross abnormalities by a technician blinded to treatment groups. For each liver sample five random sections were imaged at x 40 magnification, and scored for macrovesicular and microvesicular steatosis, where macrovesicular steatosis is defined by the vacuoles displacing the nucleus to the side, while microvesicular steatosis do not, as previously described (46). The median steatosis scores from the five sections of each animal were used to determine the median steatosis score, which was used to calculate the median for each litter, group, and age cohort.

### Epididymal Sperm Concentration and Live Dead Stain

Epididymides were transferred to an organ-well culture dish containing 500 μl of GMOPS+ culture medium at 37°C. The epididymides were punctured ten times with a 23-gauge needle (Becton Dickinson, Scoresby, VIC Australia), placed on a heated stage at 37°C and covered from light for 10 min to allow sperm to swim out (47). For each sample, a 40 µl aliquot of the sperm solution was diluted 1:10 in water (v/v) and 10 μl of this solution was pipetted onto each chamber of a haemocytometer (Neubauer, Wertheim, Germany). Sperm were counted on a Nikon Eclipse TS100 (Nikon, Tokyo, Japan) at 200 x magnification, according to previously defined methods (48). Duplicate counts were undertaken and averaged per sample prior to calculating sperm concentration by a technician blinded to treatment groups.

Live/dead sperm staining was performed based on previously described methods (49). Briefly, 10 µl of propidium iodide (0.5 mg/ml) was diluted 1:10 (v/v) with GMOPS+ culture medium and 5 µl of this solution, combined with one drop of H33342 stain (NucBlue; Thermo Fisher, Scoresby, VIC, Australia) was added to a 40 µl aliquot of sperm solution and incubated for 5 min at 37°C. Subsequently, the reaction was stopped using 5 µl of 0.1% (v/v) neutral buffered formalin (NBF) and 2 µl of bovine serum albumin (BSA; 100 mg/ml, MP Biomedical, NSW, Australia) was added to prevent sperm agglutination. The solution was centrifuged at 500 g for 5 min at room temperature and the supernatant discarded, the solution was then resuspended in 20 µl of GMOPS+ culture medium. The stained sperm solution (10 µl) was mounted on a superfrost microscope slide (Platinum Pro, Thermo Fisher) and visualised under a fluorescent microscope (Nikon Eclipse TS100) with the appropriate filters. A minimum of 10 images per sample were captured randomly using a Nikon digital sight camera. Captured images were later processed, and counted using Image J (43). Sperm nuclei stained with propidium iodide were counted as “dead”. A minimum of 200 spermatozoa were counted per slide (48). The percentage of live sperm was then calculated from these data.

### Daily Sperm Production (DSP)

Daily sperm production (DSP) was determined using a partial portion of testis, as previously described (50). Briefly, a portion of snap-frozen testis from each male was weighed and sonicated for 30 s at 22.5 kHz using a VirSonic ultra cell disruptor 100 (VirTis, Gardiner, NY, USA) in 500 µl of DSP saline buffer that contained 0.9% NaCl and 0.05% Trition-x-100 (v/v). The solution was stained with 10 µl of 0.4% trypan blue (v/v saline buffer) and vortexed for 20 s at room temperature to mix. A 20 µl aliquot of the sperm solution was diluted 1:1 with H_2_O to facilitate counting accuracy. A 10 μl volume of this solution was pipetted onto each side of a haemocytometer (Neubauer). Spermatids were counted by a technician blinded to treatment groups using light microscopy on a Nikon Eclipse TS100 (x 200) according to previously defined methods (50). Duplicate counts were undertaken and averaged for each sample. To obtain the daily sperm production, the total number of spermatids was divided by 4.84, which corresponds to the time that developing spermatids spend in steps 14 to 16 during spermatogenesis in the mouse (51) and corrected based on the weight of the original tissue portion used.

### RNA Isolation and cDNA Synthesis

A portion of testis for each male (n = 4 from different litters per group per age cohort) were homogenised and total RNA extracted using GenElute™ Mammalian total RNA miniprep kit (Sigma-Aldrich), following the manufacturer’s instructions. A section of liver from each male (n = 4 from different litters per group per age cohort) was homogenised and extracted using TRIzol reagent (Thermo Fisher) according to the manufacturer’s instruction. Extracted RNA samples were then DNase treated using Ambion TURBO DNA-free (Invitrogen, Scoresby, VIC, Australia) following the manufacturer’s specifications. Total RNA quantity and purity was assessed using the NanoDrop One/One^C^ UV-Vis Spectrophotometer (Thermo Fisher). A minimum inclusion criterion of 1.8 for the 260:280 ratio was used as a measure of quality and for samples to be used. The cDNA was synthesised from total RNA (400 ng/ml liver or 2 ng/ml testis) using the Superscript^®^III First-strand synthesis system for RT-PCR (Invitrogen) per the manufacturer’s instruction and using random hexamer primers.

### Quantitative Real Time RT-PCR

Quantitative real time RT-PCR was performed in triplicate 10 µl reaction volumes on 96 well reaction plates using SensiFAST™ SYBR Lo-ROX (Bioline, Eveleigh, Australia), as previously described (41). Both template free and minus RT triplicates were included on the plate to act as controls for genomic contamination. Reactions were run using the Viia™7 thermocycler (Applied BioSystems, Mulgrave, Australia). The genes investigated were for the liver samples: Ldlr, Acacα, Slc27a5, Pparα and Cpt1a, for the testis samples Nr5a1, Hsd17β11, Cyp19a1, and Srd5α1, with Tbp and β-actin as reference genes (see **Table 1** for a full list of gene names and primer sequences). Genes of interest were selected primarily from previously identified targets for atrazine action in both tissues, including the expression of fatty acid and cholesterol pathway members in the liver (26, 28), and steroidogenic pathway enzymes and transcription factors in the testis (20). Primers were selected from previous publications or designed using Primer Express (Thermo Fisher) and NCBI primer-BLAST (52) and were synthesised by IDT (Singapore). Primer specificity and efficiency were calculated prior to use. Cycling conditions were: 50°C for 5 min, 95°C for 10 min, then amplified for 40 cycles, where each cycle included denaturation for 15 s at 94°C, annealing for 30 s at 60°C and extension for 30 s at 72°C, followed by a final extension at 72°C for 5 min. Gene expression was quantified using Pfaffl’s (53), with Tbp as a reference gene and expressed as fold changes. Tbp was selected as the reference gene, due to consistent expression levels in treatment, individuals and cohorts.

**Table 1:** Summary of genes and primer sequences for qRT-PCR analysis of liver and testis tissues.

Gene	Accession Number	Primer Sequence		Product length
*TATA-binding protein (Tbp)*	NM_013684.3	Forward 5’- Reverse 3’-	ACGGACAACTGCGTTGATTTT ACTTAGCTGGGAAGCCCAAC	128
*Beta Actin (β-actin)*	NM_007393.5	Forward 5’- Reverse 3’-	CCACTGTCGAGTCGCGT GTCATCCATGGCGAACTGGT	91
*Acetyl- Coenzyme A carboxylase alpha (Acacα)*	NM_133360.2	Forward 5’- Reverse 3’-	TAACAGAATCGACACTGGCTGGCT ATGCTGTTCCTCAGGCTCACATCT	129
*Carnitine palmitoyltransferase 1a (Cpt1a)*	NM_013495.2	Forward 5’- Reverse 3’-	GTCAAGGTCTTCTCGGGTCG CATGCGTTGGAAGTCTCCCT	147
*Low-density lipoprotein receptor (Ldlr)*	NM_010700.3	Forward 5’- Reverse 3’-	AGGTGTGAAGATATTGACGAGTG TGAAGAGCAGATAGCCTATGGA	160
*Solute carrier family 27 (fatty acid transporter), member 5 (Slc27a5)*	NM_009512.2	Forward 5’- Reverse 3’-	AGCTATACCAGCATGTCCGC ACCAGCCGTGACTTTACCAG	115
*Peroxisome proliferator activated receptor alpha (Pparα)*	NM_001113418.1	Forward 5’- Reverse 3’-	ACGTTTGTGGCTGGTCAAGTTC GTGGGGAGAGAGGACAGATGG	125
*Cytochrome P450 family 19 subfamily A member 1 (Cyp19a1)*	NM_007810.4	Forward 5’- Reverse 3’-	CTCATTATCAGCAAGTCCTCAAGCA TAAAGAAAGGGCGAATTGTTCTCCA	163
*Hydroxysteroid (17-beta) dehydrogenase 11 (Hsd17 β11)*	NM_053262.3	Forward 5’- Reverse 3’-	CCTTGGGACGAACAGGAGTG CCCGTGCATGAGATGTTCCA	134
*Nuclear receptor subfamily 5 group A member 1 (Nr5a1)*	NM_139051.3	Forward 5’- Reverse 3’-	AGGAGGAAAGGACGATCGGA CCGCTGAACGGAAGGAGAAT	184
*Steroid 5 alpha-reductase 1 (Srd5α1)*	NM_175283.3	Forward 5’- Reverse 3’-	GTTTGCTCTGTTCACCCTGTG TGGACAGCACACTAAAGCAGG	132

### Statistical Analyses

All data were tested for normality and homogeneity using boxplots, residual plots, and the Shapiro-Wilks test. Data subjected to repeated measures analysis were tested for sphericity using the Mauchly’s sphericity test. Embryo compaction and blastocyst rates were first arc-sin transformed prior to undertaking analyses, as common undertaken for embryo development studies (54, 55). For each age cohort, body weight, cumulative weight gain, as well as food and water intake data were analysed separately and combined for each age cohort using a repeated measures ANOVA, with treatment as a fixed factor and litter as a covariate, using SAS/STAT^®^ version 9.2 (SAS Institute). All other statistical analyses were undertaken using Rstudio version 1.0.143. For each age cohort, birth weight, relative tissue weight, sperm concentration, blastocyst cell counts (ICM, TE, total, ICM:TE ratio and %ICM) and gene expression were analysed using a one-way ANOVA, with data analysed by litter where appropriate. Non-normally distributed data (survival to weaning, % live sperm, DSP and liver histology assessment) were analysed by litter using a non-parametric Mann-Whitney test. Sex ratio was compared with an expected 50:50 ratio as well as between groups by a corrected χ^2^ procedure and was double-checked by binominal analysis. Results were considered significant when *P* ≤ 0.05 and all data are expressed as the mean ± SEM, unless otherwise stated.

## Results

### Pup Sex Ratio, Birth Weight, and Survivability

There was no effect of atrazine treatment on the sex ratio of pups when compared with controls (proportion male; CON 0.64 (n = 18 male, n = 10 female), ATZ 0.50 (n = 18 male, n = 18 female; *P* > 0.1). Also, no effect of atrazine treatment compared with controls on average pup birth weight (CON 3.8 ± 0.4 g, n = 28; ATZ 4.3 ± 0.2 g, n =36; *P* > 0.1) or survival to weaning (CON n = 27/28 (96.4%); ATZ n = 34/36 (94.4%; *P* > 0.1).

### Post-Weaning Survival, and Food and Water Intake

From weaning until the end of the study, no mortality post-weaning was observed among the mice. Food and water intake were monitored for five weeks and no differences were observed between the control and treatment groups (*P* > 0.1). Average water intake for control and atrazine treatment was 25.9 ± 0.6 ml/week and 25.7 ± 0.8 ml/week, respectively, while average food intake was 27.6 ± 0.9 g/week and 27.9 ± 0.9 g/week, respectively.

### Body Weight, Relative Tissue Weights, and Morphology

**Figure 1:**
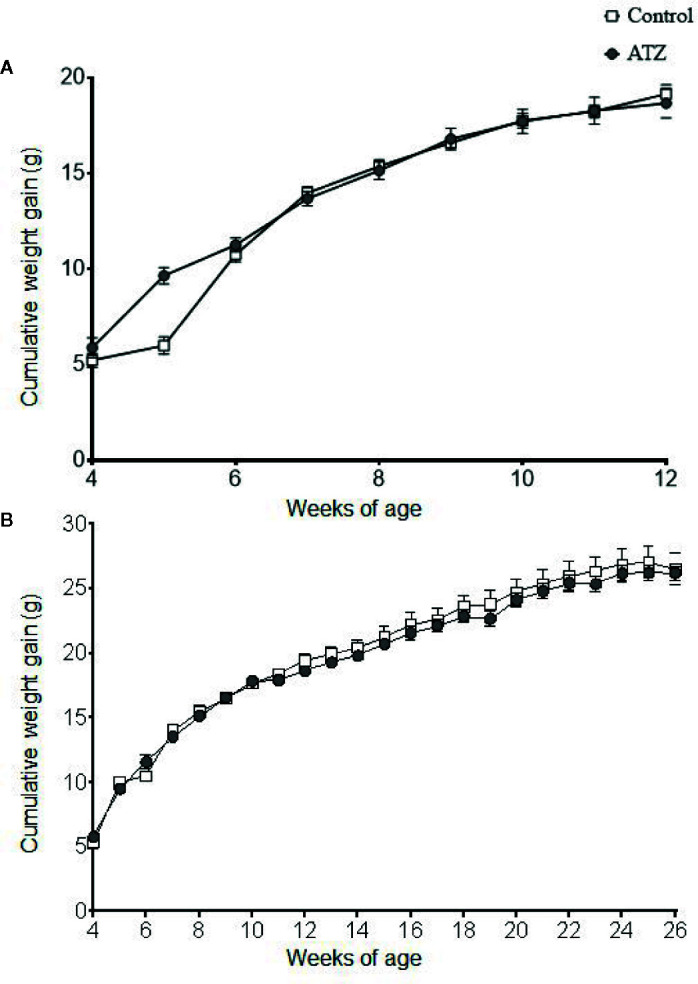
Cumulative weight gain of mice exposed to atrazine from E9.5 to **(A)** 12 weeks of age (control n = 7, atrazine n = 8) or **(B)** 26 weeks of age (control n = 10, atrazine n = 10). No significant differences were found between control and atrazine groups (P > 0.1). Data are expressed as mean ± SEM.

**Figure 2:**
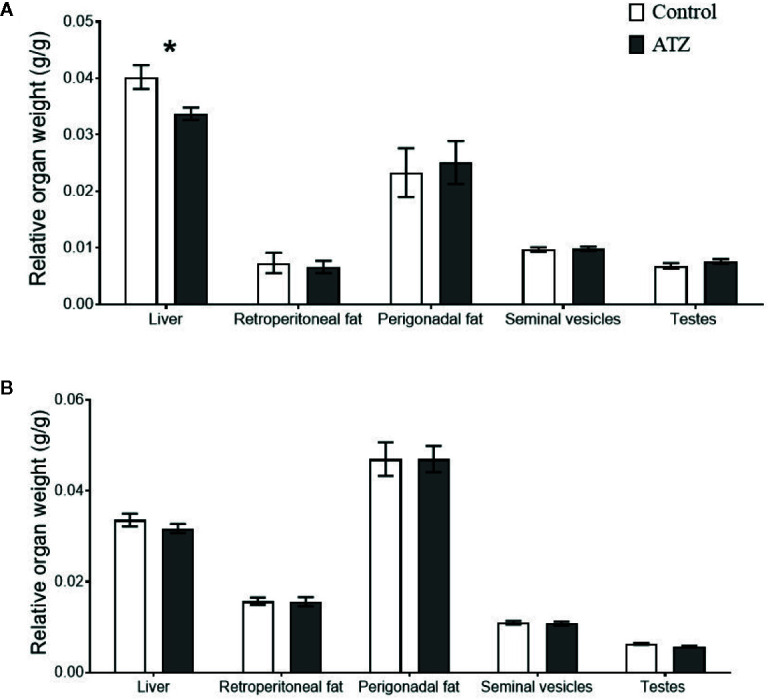
The effect of atrazine (dark grey) compared to control (white) on the relative weights of body tissue in two age cohorts. **(A)** Atrazine exposure from E9.5 to 12 weeks of age (control n = 7, atrazine n = 8), **(B)** Atrazine exposure from E9.5 to 26 weeks of age (control n = 10, atrazine n = 10). Data are expressed as mean ± SEM, *P < 0.05.

**Figure 3:**
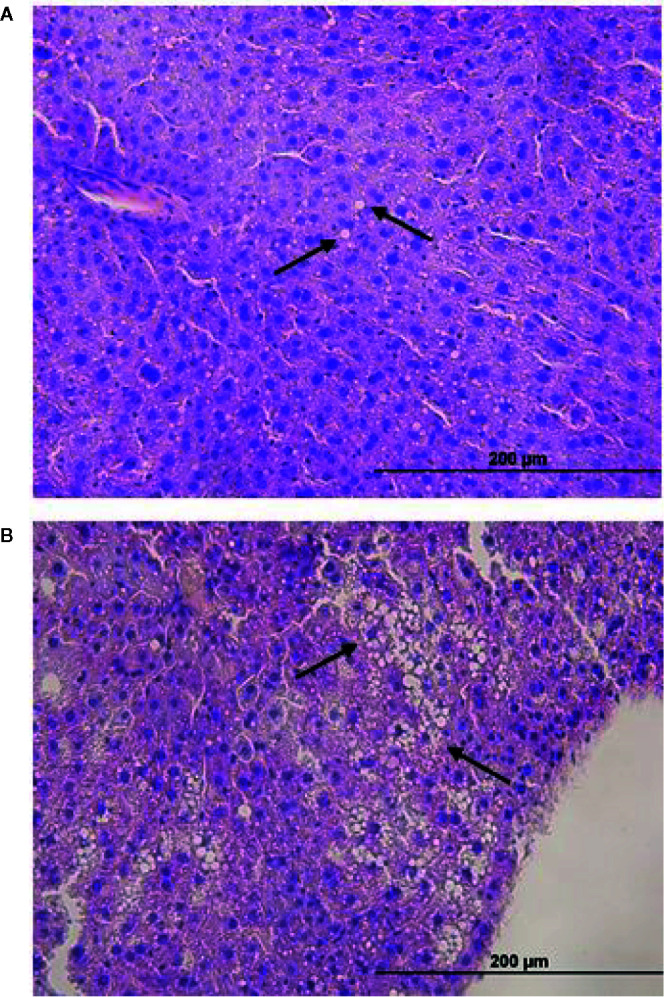
Representative H & E sections of **(A)** macrovesicular and **(B)** microvesicular steatosis in the liver (x 40 mag) of mice exposed to atrazine or control treatments from E9.5 to 12-weeks or 26-weeks of age. Arrows indicate macrovesicular and microvascular steatosis (lipid droplets).

Atrazine (5 mg/kg bw/day) exposure from prenatal E9.5 did not cause a significant increase in cumulative body weight in the 12-week (**Figure 1A**, n = 15 males from five litters, *P* > 0.1) or 26-week age cohorts (**Figure 1B**, n = 20 males from five litters, *P* > 0.1). This result was evident, irrespective of whether actual of cumulative body weight were analysed by age cohort or with 12-week and 26-week cohort data combined. In addition, there were no differences observed in the absolute or relative weights of the seminal vesicles, testes, retroperitoneal fat pads or perigonadal fat pads between the atrazine treated and the control groups in either age cohort (**Figure 2**, *P* > 0.1). However, atrazine exposure caused a significant reduction in mean absolute liver weight (CON 1.2 ± 0.06 g, ATZ 0.92 ± 0.05 g) and relative liver weight (**Figure 2A**, *P* = 0.01) compared to the control in the 12-week cohort, although this difference was not observed in the 26-week cohort (**Figure 2B**, *P* > 0.1). Visual appraisal of liver and testis sections stained with H & E revealed no changes in gross morphology in the 12- or 26-week cohorts between treatments (**Figure 3**). A more detailed study of the liver identified there was also no difference between treatments in the median score of macrovesicular or microvesicular steatosis in the livers of 12-week or 26-week cohorts (**Table 2**).

**Table 2:** The median score (25^th^ and 75^th^ percentiles) for macrovesicular and microvesicular steatosis in the livers of mice effect of atrazine exposure (5 mg/kg bw/day) on sperm characteristics.

Liver characteristics	12-week exposure cohort		26-week exposure cohort	
	Control	Atrazine	Control	Atrazine
Macrovesicular steatosis	0.0 (0.0, 0.25)	1.0 (0.0, 2.0)	1.0 (0.5, 1.0)	1.0 (0.25, 1.0)
Microvesicular steatosis	0.5 (0.0, 2.25)	1.0 (0.75, 2.0)	1.0 (0.5, 2.0)	1.0 (0.25, 1.0)

Mice were treated from E9.5 to either 12 or 26 weeks of age with atrazine (ATZ; 5 mg/kg bw/day) or control treatments. 12-week cohort (n = 6 per group), 26-week cohort (n = 8 per group).

### Sperm Parameters

We observed a significant decrease in epididymal sperm concentration of the atrazine exposed males in the 12-week cohort (**Table 3**, *P* = 0.02). However, no differences in epididymal sperm concentration were identified in the 26-week cohort (**Table 3**, *P* > 0.1). Similarly, exposure to atrazine had no effect on daily sperm production (DSP) in the testis or the percentage of live sperm in the epididymis at both 12 and 26 weeks of age (**Table 3**, *P* > 0.1).

**Table 3:** The effect of atrazine exposure (5 mg/kg bw/day) on sperm characteristics.

Sperm characteristics	12-week exposure cohort		26-week exposure cohort	
	Control	Atrazine	Control	Atrazine
Sperm concentration (M/ml)	2.53 ± 0.47	1.04 ± 0.18*	2.50 ± 0.32	2.73 ± 0.25
Daily sperm production (M/Testis)	3.61 (3.25, 4.50)	4.06 (3.81, 5.09)	4.63 (4.23, 5.02)	4.13 (3.87, 5.62)
% Live sperm	59.0 (56.2, 80.8)	63.5 (57.2, 75.4)	62.2 (60.1, 66.1)	60.9 (56.4, 67.1)

Mice were treated from E9.5 to either 12 or 26 weeks of age. *represents a significant difference between treatment groups within an age cohort (P < 0.05). Data are means ± sem or median (25^th^ percentile, 75^th^ percentile). 12-week cohort (control n = 7, atrazine n = 8), 26-week cohort (control n = 10, atrazine n = 10).

### Metabolic Gene Expression in the Liver

**Figure 4:**
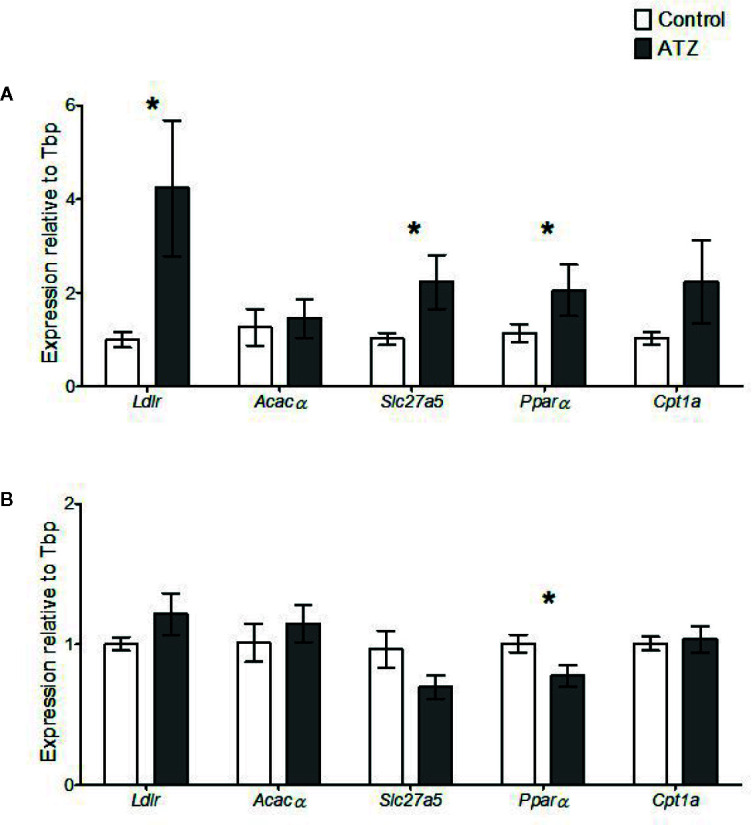
Quantitative gene expression of Low-density lipoprotein receptor (Ldlr), Acetyl- Coenzyme A carboxylase alpha (Acacα), Solute carrier family 27 (fatty acid transporter), member 5 (Slc27a5), Peroxisome proliferator activated receptor alpha (Pparα), and Carnitine palmitoyltransferase 1a (Cpt1a) in the liver. Mice were treated with control vehicle (white) or atrazine (dark gray) from E9.5 to **(A)** 12 weeks of age (n = 4) or **(B)** 26 weeks of age (n = 4). Genes are normalized to TATA box binding protein (Tbp). Significance between groups is indicated by *(P < 0.05). All data are expressed relative to Tbp ± SEM calculated using the delta delta CT method.

The expression of genes related to lipid homeostasis was analysed in the liver. Atrazine exposure from E9.5 to 12 weeks of age caused an increase in the expression of several genes involved in lipid uptake into the liver. Specifically, *Slc27a5*, which encodes fatty acid transporter protein 5, was increased in the 12-week atrazine group compared with the control (**Figure 4A**, *P* = 0.02). In addition, the expression of *Ldlr*, which encodes a protein responsible for the transport of low-density lipoproteins into the liver, was significantly increased (**Figure 4A**, *P* = 0.02). The expression of the transcriptional factor *Pparα* which is a regulator of fatty acid metabolism, in particular *Slc27a5*, was also significantly increased (*P* = 0.05) in the atrazine treated group compared to the control group (**Figure 4A**). No change in the expression of *Acacα* or *Cpt1a* was determined (**Figure 4A**, P > 0.1). In the 26-week age cohort, a significant decrease was identified in the expression of *Pparα* (**Figure 4B**, *P* = 0.03), while no differences were observed in the expression of any of the other genes studied (**Figure 4B**, *P* > 0.1).

### Steroidogenic Gene Expression in the Testis

**Figure 5:**
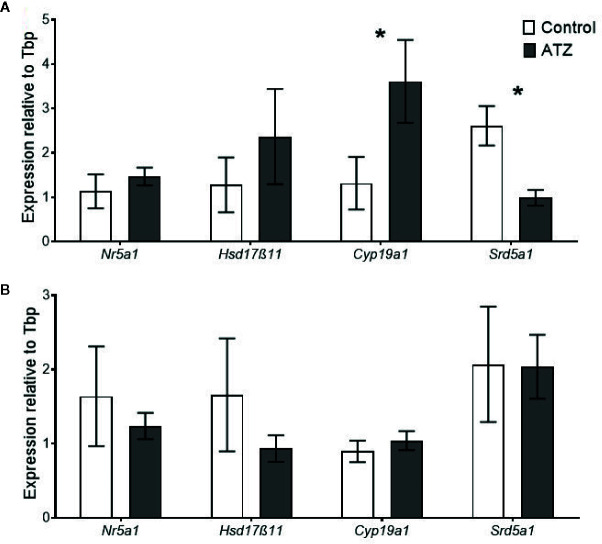
Quantitative gene expression of Nuclear receptor subfamily 5 group A member 1 (Nr5a1), Hydroxysteroid (17-beta) dehydrogenase 11 (Hsd17β11), Cytochrome P450 family 19, subfamily a, polypeptide 1 (Cyp19a1), and Steroid 5 alpha-reductase 1 (Srd5α1) in the testis. Mice were treated with either a control vehicle (white, n = 4) or atrazine (dark grey, n = 4) from E9.5 to **(A)** 12 weeks of age or **(B)** 26 weeks of age. Genes were normalised to TATA box binding protein (Tbp). Significance between groups is indicated by *P < 0.05. All data are expressed relative to Tbp ± SEM calculated using the delta delta CT method.

Exposure to atrazine from E9.5 to 12 weeks of age caused a change in the expression of steroidogenic genes in the testis. There was a significant increase in the expression of *Cyp19a1* (**Figure 5A**, *P* = 0.05), the gene that encodes for the aromatase, which is responsible for the conversion of androgens to oestrogens. Conversely, there was a decrease in the expression of *Srd5α1* (**Figure 5A**, *P* = 0.008), the gene that encodes for 5-α reductase, which is responsible for the conversion of testosterone to the more potent androgen DHT. The expression levels of *Nr5a1* and *Hsd17β11* were not found to be affected by the exposure to atrazine (**Figure 5A**, *P* > 0.1). When the exposure period to atrazine was increased to 26 weeks of age, no significant differences were found between any of these genes in the testis (**Figure 5B**, *P* < 0.1).

### Effects of Paternal Atrazine Exposure on Embryo Development

**Figure 6:**
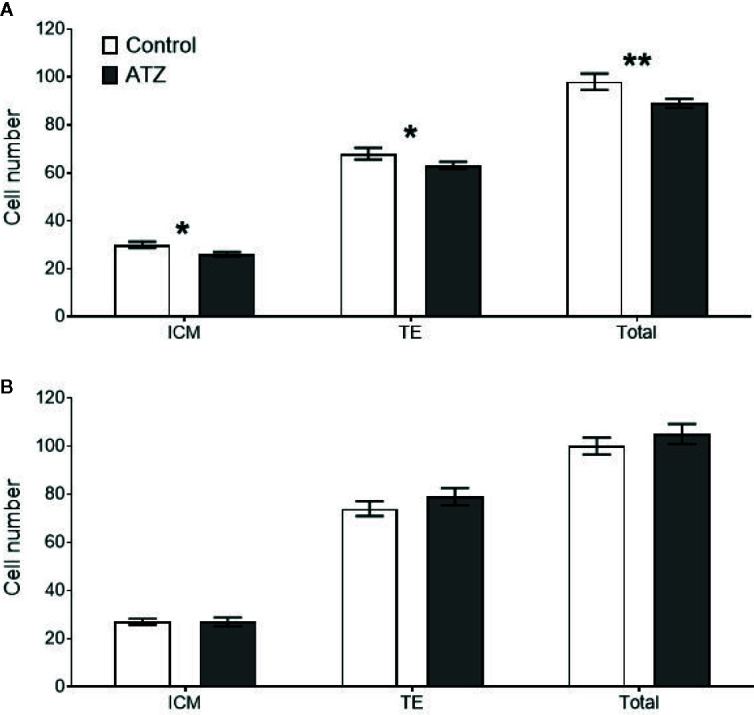
Mean cell counts of the inner cell mass (ICM), trophectoderm (TE), and total cells for all embryos that reached the blastocyst stage or above. White bars represent embryos generated from control males, and grey bars represent embryos from atrazine exposed males, n > 47 embryos per group. **(A)** Embryos derived from males exposed to atrazine or vehicle control from E9.5 to 12 weeks of age (n = 24), **(B)** Embryos derived from males exposed to atrazine or vehicle control from E9.5 to 26 weeks of age (n = 20). *represents a significant difference between groups (*P* < 0.05), **represents a significant difference (*P* = 0.01). Data are means ± SEM.

Preimplantation embryos derived from sperm of males exposed to atrazine had no effect on key embryo development parameters, with compaction and blastocysts rates unaffected in both age cohorts (**Table 4**, *P* > 0.1). Despite this, paternal atrazine exposure affected the number of cells within the preimplantation embryo. Sperm from the 12-week cohort of atrazine exposed males generated embryos with fewer ICM (*P* = 0.02), TE (*P* = 0.05) and total cells (*P* = 0.01) than the embryos generated using sperm from unexposed males (**Figure 6A**). However, in the 26-week cohort, there was no difference in cell counts of embryos generated from atrazine exposed fathers compared to the unexposed fathers (**Figure 6B**). In addition, no differences were observed between treatments in the ICM:TE ratio, or %ICM in either age cohort (*P* > 0.1, **Table 4**).

**Table 4:** The effect of paternal atrazine exposure (5 mg/kg bw/day) on the growth rate of the pre-implantation embryo and the allocation of cells to the inner cell mass (ICM) or trophectoderm (TE).

Parameter	12-week exposure cohort		26-week exposure cohort	
	Control	Atrazine	Control	Atrazine
Compaction rate (%)	73.0 ± 4.48	79.4 ± 7.70	73.3 ± 4.22	79.4 ± 7.84
Blastocyst rate (%)	70.0 ± 1.67	78.2 ± 5.76	63.4 ± 8.85	74.5 ± 4.31
ICM:TE Ratio	0.45 ± 0.02	0.42 ± 0.01	0.40 ± 0.02	0.35 ± 0.02
% ICM	30.51 ± 0.79	29.36 ± 0.75	27.71 ± 1.01	25.35 ± 1.08

Compaction rate was calculated from the number of 2PN embryos that successfully developed to compaction stage and blastocyst rates were determined by the number of 2PN stage embryos that developed to blastocyst stage. Embryonic development rates were determined per culture (n = 5 cultures per age cohort), while cell lineage was determined per embryo (n > 43 per group per age cohort). Embryos generated from males (n > 5) from each treatment group and age cohort. No Significant differences between groups within age cohort were identified (P > 0.1). Data are expressed as mean ± SEM.

## Discussion

This study identified that chronic atrazine exposure, beginning prenatally, negatively affected metabolic and reproductive parameters in young male mice, with multigenerational effects on preimplantation embryos derived from the sperm of exposed males. Specifically, atrazine exposure reduced the relative liver weight and perturbed hepatic gene expression. In addition, atrazine exposure affected steroidogenic pathway genes, *Cyp19a1* and *Srd5a1*, decreased epididymal sperm concentration and the number of cells in blastocyst stage embryos, suggesting that future offspring may be affected. Notably though, these effects were not evident when there was prolonged exposure to atrazine, into middle-age, indicating atrazine-induced changes are only evident in the growth phase of early adult life or more likely, the cellular effects of aging are greater than those resulting from atrazine exposure.

Atrazine exposure from E9.5 to 12 weeks of age caused a decrease in the relative weight of the liver and changes to metabolic gene expression, despite no change in overall body weight. Previously, rodent studies from our own group and others that assessed liver weights reported an increase or no change in liver weight, and no change in body weight (26, 31, 33). However, these rodents were exposed post weaning to atrazine, between 300 µg/kg bw/day to 5 mg/kg bw/day. In this study, the treatment of atrazine began on E9.5, which is a critical period for development in mice, when organogenesis of the liver is underway and sexual differentiation initiates (56, 57). Therefore, the reduction in liver weight and the changes in gene expression observed in atrazine treated males could be attributed to atrazine disturbing gestational hepatic development. From findings of other studies, one mechanism maybe *via* the ability of atrazine to alter mitochondrial function by interfering with complexes I and III in the electron transport chain (28). This can increase reactive oxygen species, disrupt energy metabolism and induce oxidative stress, thereby creating a sub-optimal environment for organ development (58). This is supported by studies in human liver cells that revealed atrazine exposure (0.625 µg/ml) decreased cell proliferation rates and induced mitochondrial dysfunction (59, 60). The current finding adds to the growing amount of literature that proposes early life stressors during critical periods of development can alter tissue structure and physiology (38). Surprisingly, in the 26-week cohort, no change in liver weight was observed in the atrazine treated mice. This suggests that although there may be differences in liver development, eventually the chronic dose was not high enough to permanently delay development, and the liver grows to a similar size to that of the control, as evidenced by an increase in absolute liver weights of the 26-week atrazine cohort in the current study, whilst body weight remained comparable between treatments at 12 and 26 weeks of age.

In addition to altered liver growth, the 12-week cohort showed significant changes in the expression of genes relating to hepatic lipid homeostasis. Notably, there was an increase in the expression of *Slc27a5*, which encodes for fatty acid transporter protein 5. This gene is exclusively expressed in the liver and contributes significantly to the uptake of fatty acids, and may lead to a build-up of triglycerides in the liver (61). Additionally, this study observed increases in *Ldlr* expression, a protein that transports low density lipoproteins into cells (62). Both genes are transcriptionally regulated by *Pparα (* 63, 64), which was also found to be increased in this study. Collectively, these findings support the notion that atrazine, at the current dosage, perturbs hepatic lipid and fatty acid metabolism, by increasing their uptake but not their storage, as no differences were evident in markers of liver steatosis, i.e., in the presence or size of lipid vesicles. A finding in contrast to previous studies using higher doses administered postnatally that identified an increased presence of hepatic lipid vesicles (26, 28). The effects of atrazine on hepatic metabolic gene expression at 12 weeks of age may be reflective of the altered liver size or development, although further molecular studies are required to verify the exact mechanism of action. However, in the 26-week cohort similar results were not observed. It is possible that any effects of atrazine on metabolic gene expression were masked in the 26-week cohort, due to increased ageing and a sedentary lifestyle, which themselves are potent regulators of general metabolic rate.

Alterations in metabolism can also have an indirect effect on reproduction as both systems are controlled by overlapping regulatory pathways (65). Disruptions in metabolic gene expression can alter metabolic hormones and influence the hypothalamic–pituitary–gonadal axis (65), as can exposure to atrazine (66), which both alter the production of steroid hormones. In addition, changes in lipid regulation can affect cholesterol availability, which is the precursor for sex steroid hormones (67). Subtle changes in sex steroid production can alter sperm development and lead to increased sub-fertility (2). We show that atrazine exposure directly altered steroidogenic gene expression in the testis, although the current study did not measure circulating steroid concentrations. The changes in testis gene expression are similar to the reported effects of atrazine in previous studies (23, 68) that found atrazine can impact the physiology of reproductive organs, especially in the male (20, 24). However, no changes were identified in the relative weight of the reproductive organs or histology of the testis, and this is likely due to the lower dose of atrazine utilised, since previous studies report changes with much higher doses (> 100 mg/kg bw/day) (20, 24, 69).

Atrazine reduced *Srd5α1* expression, which encodes 5α-reductase, an enzyme that converts testosterone into DHT (70). A novel aspect of the current study was to determine local changes of 5α-reductase in the testis, rather than in accessory organs that are the commonly investigated site and target for DHT activity (71, 72). This study also found atrazine exposure increased *Cyp19a1* expression, which encodes the enzyme aromatase, a finding previously reported in rodent (20) and fish studies (18), as well as in human cells lines (23). Aromatase is responsible for converting testosterone to oestradiol) (21). Changes in steroid hormone production can also affect the development of sperm (36). In the 12-week cohort, no change in the daily sperm production was identified, however, a significant reduction in the concentration of sperm from the epididymis was observed. This is consistent with the results of previous studies that used higher atrazine concentrations (> 60 mg/kg bw/day) (19, 27), suggesting that hormonal imbalances may affect the survival and maturation of sperm as it travels through the epididymis. Equally, incubation of bovine epididymal sperm *in vitro* with environmentally-relevant atrazine dosages negatively affects sperm viability, membrane disruption, mitochondrial function, and initiation of the acrosome reaction (73). In the 26-week cohort, no changes in steroidogenic gene expression or sperm viability were identified and this is likely due to the changes associated with age. During ageing, there is a progressive decline in steroidogenic machinery and sperm viability (74), therefore, subtle effects caused by atrazine would probably be masked by the effect of ageing in the older cohort.

In addition to altered sperm concentration, the blastocysts derived from sperm from the 12-week atrazine cohort had a reduction in ICM, TE and total cell numbers. This indicates that there were subtle changes occurring within the sperm that were passed on to the embryo, although the current study did not investigate these. However, in the preimplantation embryos generated from the 26-week atrazine exposed males, there were no changes to the cell numbers in the blastocysts, suggesting that atrazine was having a greater effect on the sperm of the younger males. From other studies, it is now known that the sperm can deliver a range of miRNAs, which can remain stable in the embryo until embryonic activation (75). Early life stressors are known to alter the miRNA content and epigenetic modifications in sperm (76), which can affect the embryo (75). The current study focussed on paternal exposure, starting *in utero*, through maternal exposure. Previous studies have identified maternal atrazine exposure can promote methylation changes and sperm epi-mutations in male offspring, and this can lead to increased incidence of disease in subsequent generations (77). Furthermore, atrazine may increase oxidative stress in the testis, resulting in increased DNA damage in the sperm (76). However, it is not known in the current study whether the changes in blastocyst were due to epigenetic changes or oxidative stress in the sperm, hence, further studies are required to identify the possible mechanism by which paternal atrazine exposure can affect preimplantation embryo cell number. This is important as alteration in cell number is a predictor of embryo viability (78) and is associated with long-term effects on offspring health and the health of subsequent generations (38). In rodent studies, reduced total blastocyst numbers were associated with reduced implantation rates, small prenatal growth and a disproportional growth of organs (78, 79). However, further studies using this or a more environmentally-relevant atrazine doses are required to confirm and elucidate effects on the embryo and developing foetus.

### Conclusions

This study showed that atrazine exposure, starting prenatally, caused negative metabolic and reproductive effects in adult male mice, which differed in severity depending on the duration of atrazine exposure. These effects were relatively subtle, as would be expected, based on conflicting evidence reported from human studies (15) and animals in the natural environment (3, 17). This does not detract from the importance of identifying them, with additional endpoint measures, such as determining the circulating or local steroid concentrations, would help identify possible mechanisms. The findings of this and other studies also highlight the importance of assessing the effects of atrazine at numerous life stages, as well as the effects over multiple generations. However, these measures and their transgenerational implications are generally not assessed in traditional toxicological assessments. Due to the widespread and continued use of atrazine, it is essential that the implications and health risks associated with its use are understood and that a broader range of endpoints are investigated.

## Data Availability Statement

The raw data supporting the conclusions of this article will be made available by the authors, without undue reservation.

## Ethics Statement

The animal study was reviewed and approved by The University of Melbourne Animal and Ethics committee (AEC 1513481.5).

## Author Contributions

AH undertook the study, collected the data, analysed the data, and drafted the manuscript. BF helped with the undertaking of the animal study, embryo cultures, gene expression studies, and analyses, as well as preparing the figures. MG helped analyse the data, was responsible for the experimental design, and supervised the study. All authors contributed to the article and approved the submitted version.

## Funding

This work was supported by University of Melbourne internal funds [R06000010 to MG].

## Conflict of Interest

The authors declare that the research was conducted in the absence of any commercial or financial relationships that could be construed as a potential conflict of interest.
